# Implications of Therapy-Induced Selective Autophagy on Tumor Metabolism and Survival

**DOI:** 10.1155/2012/872091

**Published:** 2012-04-05

**Authors:** Luke R. K. Hughson, Vincent I. Poon, Jaeline E. Spowart, Julian J. Lum

**Affiliations:** ^1^Deeley Research Centre, BC Cancer Agency, 2410 Lee Avenue, Victoria, BC, Canada V8R 6V5; ^2^Department of Biochemistry and Microbiology, University of Victoria, Petch Bldg, Ring Road, Victoria, BC, Canada V8P 5C2

## Abstract

Accumulating evidence indicates that therapies designed to trigger apoptosis in tumor cells cause mitochondrial depolarization, nuclear damage, and the accumulation of misfolded protein aggregates, resulting in the activation of selective forms of autophagy. These selective forms of autophagy, including mitophagy, nucleophagy, and ubiquitin-mediated autophagy, counteract apoptotic signals by removing damaged cellular structures and by reprogramming cellular energy metabolism to cope with therapeutic stress. As a result, the efficacies of numerous current cancer therapies may be improved by combining them with adjuvant treatments that exploit or disrupt key metabolic processes induced by selective forms of autophagy. Targeting these metabolic irregularities represents a promising approach to improve clinical responsiveness to cancer treatments given the inherently elevated metabolic demands of many tumor types. To what extent anticancer treatments promote selective forms of autophagy and the degree to which they influence metabolism are currently under intense scrutiny. Understanding how the activation of selective forms of autophagy influences cellular metabolism and survival provides an opportunity to target metabolic irregularities induced by these pathways as a means of augmenting current approaches for treating cancer.

## 1. Introduction

In order to evade barriers against cancer progression and treatment resistance, tumor cells undergo metabolic adaptations and develop mechanisms to resist apoptosis [1]. Apoptosis resistance in tumor cells can occur through multiple changes, none of which are mutually exclusive. For example, tumor cells enhance antiapoptotic signaling pathways and upregulate the removal or repair of damaged DNA as well as denatured proteins. Overcoming stressors that activate apoptosis requires higher rates of energy production and necessitates that tumor cells make metabolic changes to sustain antiapoptotic signaling, DNA repair mechanisms, and elevated protein turnover. While anticancer therapies that target these essential processes have been proven effective [2–4], improved outcomes may be achieved by combining them with metabolic inhibition.

Metabolic inhibitors have been shown to improve the efficacy of standard therapies in various cancer types [5–8]. Furthermore, the increase in toxicity that is achieved when metabolic inhibitors are combined with standard therapies is often well tolerated clinically, supporting the feasibility of this approach for treating cancer [9, 10]. As a result, there is a need to increase the development of therapeutic strategies that exploit key metabolic processes in tumors, while having minimal impact on normal cells. Anticancer drugs designed to activate apoptosis by causing mitochondrial depolarization, DNA damage, and misfolded protein aggregates restructure cellular metabolism in ways that could be targeted to enhance the selective killing of tumor cells. Stress induced by these drugs activates selective forms of autophagy that could play a central role in reprogramming cellular metabolism in tumor cells following exposure to anticancer therapy.

During autophagy, double-membraned vacuoles sequester bulk cytoplasm and whole organelles (so-called macroautophagy), or engulf selective cargo for degradation. In recent years, it has been discovered that autophagy selectively degrades damaged cellular constituents, such as the mitochondria (mitophagy) and portions of the nucleus (nucleophagy), as well as misfolded protein aggregates (ubiquitin-mediated autophagy), during specific types of cellular stress [11–13]. In the following sections, we review general features of autophagy as well as unique characteristics of mitophagy, nucleophagy, and ubiquitin-mediated autophagy and consider how mitochondrial depolarization, nuclear damage, and the accumulation of misfolded protein aggregates induced by anticancer agents may impact tumor cell metabolism and viability.

## 2. General Features of Autophagy

Selective forms of autophagy share many common features with macroautophagy. It should be noted that the precise localization signals and protein-protein mediators of selective autophagy have not been fully defined; however evidence suggests that structures within the cell are degraded using components of the general autophagy machinery. An elongating phagophore encapsulates cellular cargo in a double-membraned vacuole called an autophagosome and fuses with lysosomes, resulting in the hydrolytic digestion of the autophagosome contents. Permeases efflux the digested cargo from the degradative compartment into the cytosol where molecules serve as either metabolic or biosynthetic precursors.

To date, over 30 autophagy-related proteins have been reported downstream of the mammalian target of rapamycin (mTOR), a serine/threonine kinase and master regulator of autophagy [14]. When mTOR is inhibited, it ceases to negatively regulate autophagy [15, 16]. Central to the autophagy pathway is the Beclin1/Vps34 (phosphatidyl-inositol-3 kinase (PI3K) class III) complex, the ULK complex, and two ubiquitin-like systems: the Atg12-Atg5 conjugation system and the Atg8/microtubule-associated protein 1 light chain 3 (LC3) conjugation system [17–21]. In addition, other factors, such as Atg9L1, appear to be indispensable for autophagy to occur [22]. There are likely other converging pathways required for induction of autophagy and these may be context dependent. Collectively, these components appear to play integral roles in mediating autophagosome formation, elongation, and closure [17–21]. For a detailed discussion of the autophagy signal transduction cascade, the reader is directed to several recent reviews [23, 24]. Here, we focus on key components of the general autophagic machinery and consider how they interact with unique factors associated with various forms of selective autophagy.

## 3. Mitophagy

### 3.1. The Mitophagy Pathway

**Figure 1:**
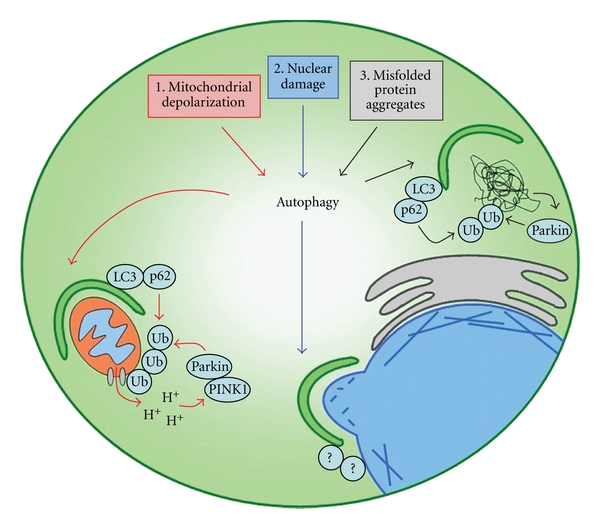
Anticancer agents may activate selective forms of autophagy by causing ΔΨm depolarization, nuclear damage, and misfolded protein aggregates. (1) Drugs that open mPTPs are known to cause ΔΨm depolarization, which may result in the recruitment of PINK1 and Parkin. It is hypothesized that this would promote mitochondrial polyubiquitination and selective targeting to the autophagosome through the LC3:ubiquitin adapter proteins, such as p62. (2) DNA damaging agents may promote the selective autophagy of structurally damaged portions of nuclei in mammals in a process dependent on the cleavage of lamin and emerin intermediate filaments in the nuclear periplasm. To date, the mammalian adapter proteins that target the autophagosome to the nucleus have not been identified. (3) Drugs that inhibit the proteosome are known to cause an accumulation of misfolded protein aggregates in tumor cells, which results in Parkin mediated polyubiquitination and targeting to the autophagosome through p62.

While studying the process of organelle turnover, it was assumed that autophagic degradation of mitochondria was a random process because autophagosomes were observed to contain a variety of cytoplasmic components including proteins, endoplasmic reticulum, peroxisomes, and mitochondria [25]. However, recent evidence suggests that autophagic digestion of mitochondria is a selective process [26] (Figure 1). One way that mitophagy can be induced is by the opening of mitochondrial membrane permeability transition pores (mPTP) and the depolarization of the electrochemical proton gradient across the inner mitochondrial membrane [11, 27, 28]. Following mitochondrial membrane potential (ΔΨm) depolarization, PTEN-induced putative kinase 1 (PINK1) localizes to the mitochondria and recruits Parkin, an E3 ubiquitin ligase that forms polyubiquitin chains linked through K27 and K63 on voltage-dependent anion channel (VDAC) proteins on the outer membrane of mitochondria [28]. These polyubiquitin chains appear to serve two purposes: first, to tether clusters of dysfunctional mitochondria together and second, to target these structures for autophagic degradation [28]. Interestingly, both K27 and K63 polyubiquitin linkages have been correlated with lysosomal localization and/or autophagic degradation of proteins [28–30]. These linkages differ from the canonical G76–K48 ubiquitin linkages characteristic of proteins destined for proteasomal degradation [31, 32], supporting the hypothesis that site-specific ubiquitination targets mitochondria for selective autophagic degradation.

The mechanisms responsible for ushering ubiquitinated mitochondria to the nascent phagophore for autophagic degradation are controversial. Initially, Geisler et al. proposed p62 to be the principle mediator of crosstalk between the selective and degradative machinery of mitophagy, as silencing of p62 was observed to inhibit the degradation of mitochondria, polyubiquitin, and Parkin but not the colocalization of these structures following ΔΨm depolarization [28]. This hypothesis is supported by evidence demonstrating that p62 binds K63-linked polyubiquitin [33] as well as the lipidated form of the autophagosome bound protein, LC3 [34], which plays an important role in autophagosome formation and closure [35]. However, contrary to the results of Geisler et al., two recent studies have independently demonstrated that p62 is essential for clustering but not degradation of depolarized mitochondria [36, 37]. These disparate results are difficult to reconcile given that the investigators used the same cell types and siRNAs in their respective studies [28, 36]. However, the existence of p62-independent mitophagy does not exclude the possibility that multiple adapter molecules capable of binding polyubiquitin and LC3 such as p62, Nrb1, and Nix function redundantly to bridge the selective and degradative machinery of mitophagy [36, 37].

In addition to the selective machinery described above, the process of mitophagy also employs conventional proteins associated with macroautophagy and thus can be blocked pharmacologically with general autophagy inhibiting drugs such as chloroquine, 3-methyladenine, and wortmannin [38, 39]. These drugs are commonly used inhibitors of lysosomal acidification and autophagy inducing signals generated by class III PI3Ks. To date, it remains unclear how the autophagic machinery is activated in concert with PINK1, Parkin, and p62 during ΔΨm depolarization. One possibility is that Parkin stimulates the generation of autophagy inducing signals from the Beclin1/Vps34 class III PI3K complex by interacting with the autophagy promoting protein, Ambra1 [40]. In addition, given that mitochondria are responsible for maintaining the majority of cellular adenosine triphosphate (ATP) pools, it is likely that energy sensors detecting increases in the intracellular ratio of adenosine monophosphate (AMP): ATP, such as 5′ AMP-activated protein kinase (AMPK), activate autophagy during ΔΨm depolarization.

### 3.2. Mitophagy Inducing Signals Are Generated by Anticancer Agents

Many ionophores including carbonyl cyanide m-chlorophenylhydrazone (CCCP), p-trifluoromethoxy carbonyl cyanide phenylhydrazone (FCCP), 2,4-dinitrophenol, and fluoride curcumin derivatives have been demonstrated to induce mitophagy by causing ΔΨm depolarization [28, 41, 42]. In addition to activating mitophagy, these uncoupling agents and numerous other drugs that open mPTPs cause mitochondrial swelling and depolarization, signaling for the induction of apoptosis [43]. Among these mPTP targeting drugs are several clinically used anticancer agents, including 1-*β*-D-arabinofuranosylcytosine, butyrate, doxorubicin, etoposide, lonidamine, paclitaxel, and vinorelbine (Table 1). Drugs targeting mPTPs are attractive for cancer therapy because they mediate cytochrome c release, a potent apoptotic trigger [43]. While some of these drugs have been reported to activate autophagy, their ability to induce mitophagy specifically has not been investigated. Given that mitochondrial depolarization is a powerful inducer of mitophagy as well as apoptosis, further work must be done to determine whether mPTP targeting drugs do in fact activate mitophagy and how this impacts cellular viability.

### 3.3. Implications of Mitophagy on Tumor Cell Metabolism and Survival during Therapeutic Assault

**Figure 2:**
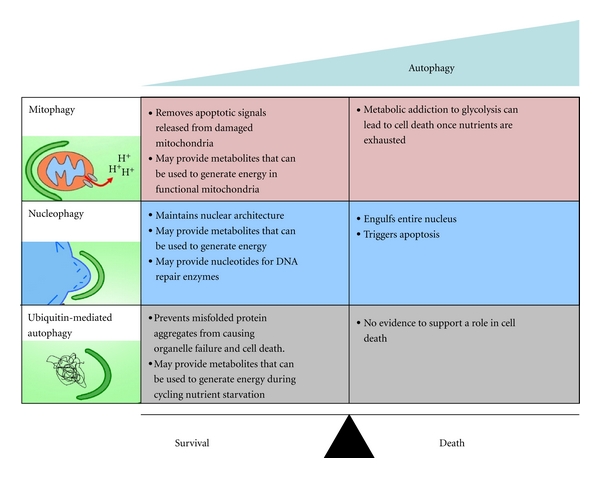
Mitophagy, nucleophagy, and ubiquitin-mediated autophagy are associated with cell survival or cell death depending on the level of activation. A homeostatic level of mitophagy promotes cell survival by liberating nutrients and by clearing dysfunctional mitochondria that signal for apoptosis. Conversely, hyperactivation of mitophagy can lead to a loss in the cell's ability to generate ATP, resulting in cell death. Similarly, a homeostatic level of nucleophagy protects cells against the accumulation of structural damage to the nucleus and may provide energetic and biosynthetic resources that aid in repair. Nucleophagy also appears to be associated with cell death in specialized cell types facing extreme stress. Ubiquitin-mediated autophagy appears to function solely as a survival pathway that clears misfolded protein aggregates and liberates metabolites that may be used for energy production.

The fate of cells that undergo ΔΨm depolarization is dependent on a variety of factors including the level of apoptotic signaling from the mitochondria and cellular metabolism. The degradation of dysfunctional mitochondria by mitophagy promotes cell survival by preventing the production and release of toxic byproducts such as reactive oxygen species and cytochrome c that signal for apoptosis [43, 89]. However, the bioenergetic consequences of mitophagy on cellular viability are more complex. On one hand, homeostatic levels of mitophagy may promote cell survival by liberating metabolites that can be oxidized in functional mitochondria for energy. Conversely, hyperactivation of mitophagy renders cells either incapable of meeting energetic demands or solely dependent upon glycolytic substrates for survival [41]. Given that many tumors are inherently dependent on aerobic glycolysis for bioenergetics (so-called Warburg effect) [90, 91], hyperactivation of mitophagy would solidify their glycolytic addiction by diverting the flux of metabolites away from the mitochondria. Evidence in support of this hypothesis has been demonstrated in HeLa cells, a human tumor cell line that does not endogenously express Parkin, and thus cannot undergo ΔΨm depolarization-induced mitophagy [41]. When HeLa cells are pretreated with the ΔΨm depolarizing agent, CCCP, the cells survive, presumably because they are able to utilize amino acids and other metabolites in the mitochondria to generate energy [41]. However, HeLa cells pretreated with CCCP and transfected with Parkin do not survive glucose withdrawal because their mitochondria are degraded by mitophagy, preventing oxidative metabolic pathways from sustaining energy pools [41]. In this model, it appears that Parkin-dependent mitophagy may promote survival by coordinating a metabolic shift from oxidative phosphorylation to glycolysis when mitochondria become dysfunctional. However, when cells undergo excessive mitophagy, the nutrient environment of the cell dictates whether cells will survive or succumb to energy crisis followed by cell death (Figure 2).

This finding may have important implications for chemotherapeutic strategies for treating some cancers. For example, administration of glycolytic inhibitors in combination with mitophagy inducing chemotherapies may potentiate killing of tumor cells as a result of increased tumor cell dependency on glycolysis following excessive mitophagy. This may explain why the efficacies of several chemotherapies that result in mPTP opening, such as paclitaxel and doxorubicin, are enhanced significantly when administered with lonidamine, a combinatorial hexokinase inhibitor and mPTP opener [92, 93].

## 4. Nucleophagy

### 4.1. Nucleophagy Pathway in Saccharomyces cerevisiae and Mammals

Maintaining proper structure, organization, and dynamics of the nucleus is essential for the vitality of most cell types [94]. Emerging evidence suggests that the selective digestion of portions of the nucleus by autophagy plays a central role in upholding nuclear integrity when structural damage occurs [12]. While nucleophagy in mammalian cells has recently been reported [12, 95–97], this process has primarily been described in yeast [94]. In most yeast models, nutrient deprivation is the stressor of choice used to induce nucleophagy [98, 99]. Following nutrient deprivation, junctions between nuclei and vacuoles (the yeast lytic compartment) are seen to increase in surface area as a result of interactions between Nvj1p, an outer nuclear membrane protein, and Vac8p, a vacuolar membrane protein [98]. Within nucleus-vacuole junctions, the nuclear envelope begins to form bulges and blebs that pinch off and are sequestered in the vacuole for degradation [99]. This gradual degradation of nuclear content is referred to as piecemeal microautophagy of the nucleus (PMN) [99]. In contrast to macroautophagy, PMN does not involve the formation of autophagosomes to envelop content to be degraded [99]. However, it has been shown that nucleophagy in yeast requires macroautophagic machinery, including the two ubiquitin-like conjugation systems and the PI3K class III complex, to mediate terminal vacuole enclosure and fusion stages [100].

In contrast to PMN seen in yeast, mammalian cells undergoing nucleophagy are able to form large autophagosomes characteristic of macroautophagy [12, 95–97]. These large double-membraned macroautophagosomes are observed to colocalize with LC3 and have been seen to envelop large portions of structurally deformed nuclei as well as small nuclear blebs [12]. While mammalian orthologs of nucleophagy adapters such as Nvj1p and Vac8p have not yet been identified, it has been shown that mutations in A-type lamins and emerin in Lmna^H222P/H222P^ mouse embryonic fibroblasts cause structural deformations in the nuclear envelope resulting in the induction of nucleophagy, possibly through similar selective adapter proteins to those described in yeast [12] (Figure 1).

### 4.2. Nucleophagy Inducing Signals Are Generated by Anticancer Agents

Cancer treatment regimens often include DNA-damaging agents in an attempt to target the nuclear content of rapidly dividing cells. One feature of many DNA-damaging agents is their activation of caspases that disassemble the nuclear lamina by cleaving lamin intermediate protein filaments. While lamin cleavage is known to increase nuclear envelope plasticity and contribute to nuclear blebbing during apoptosis [101], there is evidence that it also activates nucleophagy. Park et al. demonstrated that mutated lamins lead to deformations in the nuclear envelope that induced nucleophagy [12]. Therefore, DNA-damaging agents that disrupt the nuclear envelope may be predicted to have a similar effect. Some clinically used anticancer agents that induce DNA damage and lamin cleavage include etoposide, camptothecin, cisplatin, and 1-*β*-d-arabinofuranosylcytosine [102] (Table 1). In addition to these drugs, the cation vanadyl(IV) has been confirmed to activate oxidative stress and DNA damage resulting in nucleophagy of whole chromosomes in mitotic cells [96, 97]. While the exact mechanism of nucleophagy induction following vanadyl(IV) exposure has not been elucidated, it is possible that it may involve a similar lamin-dependent mechanism.

### 4.3. Implications of Nucleophagy on Tumor Cell Metabolism and Survival during Therapeutic Assault

Activation of nucleophagy appears to be a double-edged sword. In some models, activation of nucleophagy in response to DNA damage has been demonstrated to promote cell death by degrading whole chromosomes in oxidatively stressed mitotic cells [96, 97]. Similarly, whole autophagic degradation of the nucleus has been seen in protozoans such as *Tetrahymena thermophila*, leading to programmed cell death, albeit through different autophagic machinery than what is observed in yeast or mammals [103]. In contrast, nucleophagy has also been demonstrated to promote survival in mammalian cells by maintaining nuclear structure, and possibly through the release of nutrients for energy production [12]. To date, nucleophagy has yet to be defined in tumor cells. However, several DNA damaging anticancer agents may induce nucleophagy in tumors cells as a result of their ability to cause cleavage of lamin filaments [102] (Table 1). Some of these drugs have also been shown to facilitate a cytoprotective, autophagy-dependent surge of ATP [59], raising the possibility that nucleophagy contributes in mediating this ATP surge. It may be that ATP pools are utilized to fuel the energy costly process of DNA repair, the perpetuation of nucleophagy, or both. In addition, liberation of nucleic acids through the nucleophagic degradation of damaged DNA may contribute to increased rates of DNA repair by providing substrate for DNA repair enzymes. To counteract the potential protective role of nucleophagy, anticancer agents that induce this process could be combined with inhibitors of amino acid or lipid catabolism (major macromolecules associated with the nuclear envelope) or inhibitors of nucleophagy itself. However, given the controversial role of nucleophagy in promoting cell survival and cell death (Figure 2), further consideration must be given to systemic inhibition of nucleophagy for cancer therapy.

Another caveat to the systemic inhibition of nucleophagy is that this may lead to off-target toxicity in normal tissue. By removing the potential survival advantage imparted by nucleophagy, normal cells with DNA damage caused by nonspecific therapeutics may succumb to normal apoptotic pathways. In addition, long-lived cells such as neuronal tissue or immunological memory cells may require nucleophagy for normal maintenance of nuclear structure. Dysregulation of this process may lead to unforeseen toxicities in these cell types.

## 5. Ubiquitin-Mediated Autophagy

### 5.1. Ubiquitin-Mediated Autophagy Pathway

Tumor cells inherently have high levels of misfolded proteins due to rapid proliferation and increased intracellular acidification caused by lactic acid production during glycolysis [104, 105]. In response to misfolded proteins, cells have been shown to upregulate molecular chaperones that promote refolding of denatured proteins, proteasomal degradation of soluble misfolded proteins, and ubiquitin-mediated autophagy of protein aggregates [106–108]. The first line of defense against an aggregation of misfolded proteins is the activation of molecular chaperones of the heat shock protein family, which shield hydrophobic surfaces of denatured proteins to aid in restoration of proper folding [106]. If denatured proteins persist, ubiquitin-mediated autophagy is activated [13] (Figure 1). This process requires an intact microtubule cytoskeleton and the cytoplasmic deacetylase, histone deacetylase 6 (HDAC6), presumably to coordinate the transport of protein aggregates, autophagic machinery, and lysosomes [109, 110]. Protein aggregates are subsequently polyubiquitinated through K63 linkages by E3 ubiquitin ligases, such as Parkin [111]. This promotes the recruitment of the autophagosome adapter protein, p62, resulting in selective autophagic degradation [111, 112]. Similar to mitophagy, it appears that K63 linked polyubiquitination selectively targets misfolded proteins to the autophagosome, while crosstalk with the degradative autophagic machinery is mediated through adapters such as p62.

The general autophagic machinery appears to be activated in concert with the selective apparatus of ubiquitin-mediated autophagy by a variety of mechanisms. Following an accumulation of unfolded proteins, activating transcription factor 4 (ATF4) is stabilized, which promotes the activation of autophagy by increasing the transcription of LC3 [81, 113]. In addition, signaling from the IRE1-c-Jun NH(2)-terminal kinase pathway has been shown to be necessary for the activation of autophagy in response to proteasome inhibition [82]. Therefore, it appears that the general autophagy pathway is activated through convergent mechanisms in response to unfolded proteins.

### 5.2. Ubiquitin-Mediated Autophagy Is Induced by Anticancer Agents

The observation that tumor cells have elevated levels of misfolded proteins, and thus protein turnover, has stimulated interest in targeting components of the proteasome in order to induce proteotoxic stress in tumor cells [114]. Proteotoxicity refers to molecular damage caused by misfolded protein aggregates that can lead to organelle dysfunction and cell death [115]. The most well-known inhibitor of the proteasome, bortezomib (or Velcade(TM)), has been tested in numerous recent clinical trials and is now commonly used for the treatment of multiple myeloma and mantle cell lymphoma [84, 85]. Bortezomib has also shown some promise in other cancers, such as prostate cancer and non-small-cell lung cancer [116, 117]. Since bortezomib and other proteasome inhibitors, such as NPI-0052, compromise the cell's ability to dispose of misfolded proteins, proteasome inhibition can upregulate ubiquitin-mediated autophagy of misfolded protein aggregates as a compensatory strategy [109, 110, 118] (Table 1). Given that autophagy helps cells to degrade misfolded protein aggregates caused by proteasomal inhibition [86], it is not surprising that preclinical studies have reported increased efficacy of proteasomal inhibitors when coupled with autophagy inhibitors in colon, prostate, and breast cancer cell types [81–83]. The potential for increased therapeutic efficacy of proteasomal inhibition when combined with autophagy inhibition has even led to the initiation of a clinical trial combining bortezomib with the autophagy blocking drug, chloroquine, for the treatment of multiple myeloma (NCT01438177).

### 5.3. Implications for Ubiquitin-Mediated Autophagy on Tumor Cell Metabolism and Survival during Therapeutic Assault

In contrast to mitophagy and nucleophagy, it is unclear how the selective autophagy of misfolded proteins may restructure tumor cell metabolism. Unlike mitophagy, this process does not appear to skew nutrient utilization toward any particular pathway. Furthermore, there is no evidence that the ubiquitin-mediated autophagy of misfolded proteins promotes bioenergetics in ways similar to nucleophagy. On the contrary, the fact that tumor cells are capable of sustaining the energetically costly process of protein translation to the point where misfolded proteins aggregate and become toxic indicates that cells undergoing this type of stress are not lacking intracellular energetic resources. Collectively, these observations suggest that the ubiquitin-mediated autophagy of misfolded proteins is activated solely to remove harmful intracellular structures out of necessity. However, considering the dynamic metabolic milieu found in the tumor microenvironment, there may be a yet undefined metabolic advantage to this process over prolonged periods of time.

As a result of rapid tumor cell proliferation and fluctuations in local vasculature supplying nutrients to the tumor bed, cancer cells often undergo cycling periods of hypoxia, and presumably starvation [119]. In order to fuel essential cellular processes in the absence of exogenous metabolites, the selective autophagy of misfolded proteins may provide an internal reserve of nutrients that can be utilized during cycles of nutrient withdrawal. Therefore, blocking amino acid catabolism in tumors *in vivo* may prove to be an efficacious adjuvant to proteasome inhibition.

## 6. Conclusion

To date, anticancer agents that nonspecifically target rapidly proliferating cells remain the best treatment option for many forms of cancer. In order to ensure complete killing of tumor cells, patients are sometimes maintained on these drugs for years at a time, increasing the probability of harmful side effects. Consequently, there is a need to develop strategies to enhance the toxicity of these drugs with greater specificity towards tumor cells so that lower doses of cancer therapeutics can be administered for shorter periods of time with the same or better antitumor effect. 

Irregular metabolism is a fundamental hallmark of nearly all cancerous cells [1]. Therefore, finding ways to exploit unique metabolic adaptations and irregularities induced by anticancer agents in tumors may prove to be an effective adjuvant to standard therapies. The activation of selective forms of autophagy that degrade metabolically significant structures such as mitochondria, nuclei, and proteins may be one feature of tumors that can be exploited to cripple tumor survival.

## Figures and Tables

**Table 1:** Clinically used anticancer agents that may induce mitophagy, nucleophagy, and ubiquitin-mediated autophagy in tumor cells.

Drug	Mechanism of action	Cancer type	Confirmed autophagy inducer
Mitophagy			
1-*β*-D-arabinofuranosylcytosine	DNA synthesis inhibitor [44, 45], mPTP opener [46]	Leukemia [47], lymphoma [48]	no
Butyrate	mPTP opener [49, 50]	Leukemia [51]	yes [50]
Doxorubicin	mPTP opener [46]	Breast [52], lung, melanoma, sarcoma [53]	yes [54]
Etoposide	Topoisomerase inhibitor [55], mPTP opener [46]	Gastric [56], Kaposi's sarcoma [57], lung [58]	yes [59]
Lonidamine	Hexokinase inhibitor [60], mPTP opener [61]	Brain, lung, ovarian [60]	no
Paclitaxel	Microtubule stabilizer [62], mPTP opener [63]	Breast [64], head and neck [65], Kaposi's sarcoma [66], lung [67], ovarian [68]	yes [69]
Vinorelbine	Microtubule formation inhibitor, mPTP opener [70]	Breast [71], lung [72]	yes [73]

Nucleophagy			
1-*β*-D-arabinofuranosylcytosine	DNA synthesis inhibitor [44, 45], mPTP opener [46]	Leukemia [47], lymphoma [48]	no
Camptothecin	Topoisomerase inhibitor [74]	Gastric [75], lung [76], pancreatic [77]	yes [78]
Cisplatin	DNA intercalating agent [79]	Ovarian [68], lung [68]	yes [80]
Etoposide	Topoisomerase inhibitor [55], mPTP opener [46]	Lung [58], gastric [56], Kaposi's sarcoma [57]	yes [59]

Ubiquitin-mediated autophagy			
Bortezomib	Proteasome inhibitor [81–83]	Mantle cell lymphoma [84], multiple myeloma [85]	yes [81–83]
NPI-0052	Proteasome inhibitor [86]	Leukemia [87], multiple myeloma [88]	yes [86]

## Abbreviations

AMP: Adenosine monophosphate
AMPK: 5′ AMP-activated protein kinase
ATF4: Activating transcription factor 4
ATP: Adenosine triphosphate
CCCP: Carbonyl cyanide m-chlorophenylhydrazone
FCCP: p-trifluoromethoxy carbonyl cyanide phenylhydrazone
HDAC6: Histone deacetylase 6
LC3: Microtubule-associated protein 1 light chain 3
mPTP: Mitochondrial permeability transition pore
mTOR: Mammalian target of rapamycin
PI3K: Phosphatidyl-inositol-3 kinase
PINK1: PTEN-induced putative kinase 1
PMN: Piecemeal microautophagy of the nucleus
VDAC: Voltage-dependent anion channel
ΔΨm: Mitochondrial membrane potential.
